# A Vector with a Single Promoter for *In Vitro* Transcription and Mammalian Cell Expression of CRISPR gRNAs

**DOI:** 10.1371/journal.pone.0148362

**Published:** 2016-02-05

**Authors:** Peter J. Romanienko, Joseph Giacalone, Joanne Ingenito, Yijie Wang, Mayumi Isaka, Thomas Johnson, Yun You, Willie H. Mark

**Affiliations:** 1 Mouse Genetics Core Facility, Sloan Kettering Institute, Memorial Sloan Kettering Cancer Center, New York, NY, United States of America; 2 Developmental Biology Program, Sloan Kettering Institute, Memorial Sloan Kettering Cancer Center, New York, NY, United States of America; Osaka University, JAPAN

## Abstract

The genomes of more than 50 organisms have now been manipulated due to rapid advancement of gene editing technology. One way to perform gene editing in the mouse using the CRISPR/CAS system, guide RNA (gRNA) and CAS9 mRNA transcribed *in vitro* are microinjected into fertilized eggs that are then allowed to develop to term. As a rule, gRNAs are tested first in tissue culture cells and the one with the highest locus-specific cleavage activity is chosen for microinjection. For cell transfections, gRNAs are typically expressed using the human U6 promoter (hU6). However, gRNAs for microinjection into zygotes are obtained by *in vitro* transcription from a T7 bacteriophage promoter in a separate plasmid vector. Here, we describe the design and construction of a combined U6T7 hybrid promoter from which the same gRNA sequence can be expressed. An expression vector containing such a hybrid promoter can now be used to generate gRNA for testing in mammalian cells as well as for microinjection purposes. The gRNAs expressed and transcribed from this vector are found to be functional in cells as well as in mice.

## Introduction

Gene editing has become a valuable tool in the study of gene function across many species [1]. Currently, the most widely used approach is the CRISPR/CAS9 system derived from the adaptive immune response of bacteria [2]. To achieve genome modification by this technique, only two simple components are required: the CAS9 protein and a guide RNA (gRNA) containing 17–20 nucleotides of identity to a target sequence proximal to a protospacer adjacent motif (PAM). When gRNA forms a complex with the CAS9 nuclease, a DNA double strand break (DSB) will occur in the genomic target specified by the gRNA. For genomic manipulation of cells in culture, they are typically transfected with a single plasmid vector expressing the gRNA from an RNA Polymerase III promoter derived from the human snRNA U6 gene and the CAS9 mRNA from an RNA Polymerase II promoter [3]. Alternatively, individual plasmids carrying the gRNA template and the CAS9 gene can be co-transfected into target cells for gene editing. Recently, with CAS9 protein available from commercial sources, *in vitro* transcribed gRNAs are mixed with the purified CAS9 protein and the gRNA-CAS9 complex introduced into cells for gene editing [4].

To perform gene editing in mice, the most common procedure is to microinject gRNA and CAS9 mRNA or protein into the pronucleus or the cytoplasm of the zygote. In this approach, the gRNA is generally transcribed *in vitro* from a T7 bacteriophage promoter [5]. Alternatively, a plasmid expressing a gRNA from an hU6 promoter and Cas9 can be injected into the mouse zygote [6]. The CAS9 protein can also be complexed with *in vitro* transcribed gRNA then directly injected into the zygote to generate insertions or deletions (INDELs) [7] or to perform gene modification [8]. Prior to pronuclear injection, most laboratories test the activity of a gRNA by transfecting tissue culture cells with plasmids that express gRNA from a vector-based hU6 gene promoter along with Cas9 and determine whether INDELs are generated at the specified locus. To produce gRNA for microinjection and for cell transfections, it is necessary to make two separate plasmid vectors, one expressing the gRNA from a T7 promoter and the other from the hU6 promoter. Alternatively, without additional cloning one can use PCR and appropriate primers to synthesize an amplicon containing the same gRNA sequence with T7 added as the promoter and then perform *in vitro* transcription rather than make a new vector [9].

To avoid these multiple cloning steps, we have constructed a plasmid vector containing a single U6T7 hybrid promoter with an adjacent cloning site for insertion of a gRNA template. In this report, we show that this vector is capable of producing gRNAs by both the hU6 and the T7 promoters. When transfected into mouse cells in conjunction with a CAS9 expression vector, the U6T7 hybrid promoter expresses a functional gRNA as INDELs are detected at the genomic target site. When the T7 polymerase is used to transcribe the gRNA template *in vitro*, the gRNA injected into mouse zygotes produced the expected gene modification in embryos and the animals.

## Materials and Methods

### Construction of the U6T7 hybrid promoter

The U6T7 hybrid promoter together with a gRNA scaffold was synthesized as a 373 bp product (Genscript) with the following sequence: AAGGTCGGGCAGGAAGAGGGCCTATTTCCCATGATTCCTTCATATTTGCATATACGATACAAGGCTGTTAGAGAGATAATTAGAATTAATTTGACTGTAAACACAAAGATATTAGTACAAAATACGTGACGTAGAAAGTAATAATTTCTTGGGTAGTTTGCAGTTTTAAAATTATGTTTTAAAATGGACTATCATATGCTTACCGTAACTTGAAAGTATTTCGATTTCTTGGCTTTATATATCTTGCTAATACGACTCACTATAGGAGAGACCGAGAGAGGGTCTCAGTTT*TAGAGCTAGAAATAGCAAGTTAAAATAAGGCTAGTCCGTTATCAACTTGAAAAAGTGGCACCGAGTCGGTGCTTTT***TTTAAA**

The fusion fragment was placed into the pUC57-Kan vector (Genscript) at the XbaI cloning site and the plasmid was designated pU6T7. Specific gRNAs can be inserted downstream from the U6T7 hybrid promoter using the overhangs (underlined) generated by BsaI digestion. The gRNA scaffold is indicated by italics. Linearizing the pU6T7 plasmid by digestion with DraI (bold) will permit transcription *in vitro* of the gRNA from the T7 promoter.

**Fig 1:**
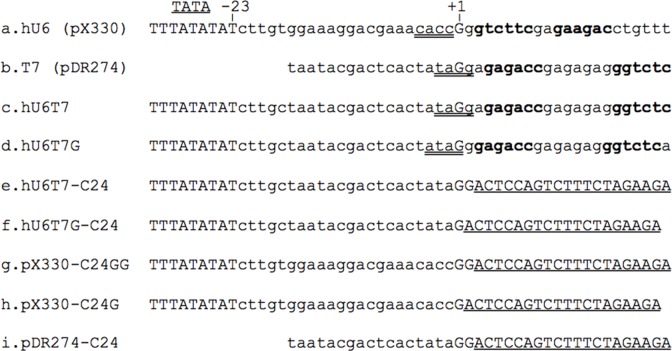
Sequences of hU6, T7 and U6T7 hybrid promoters. Sequence comparison of the hU6, T7 and the two bifunctional U6T7 and U6T7G hybrid promoters. (a) human U6 promoter (from plasmid pX330), (b) bacteriophage T7 promoter (from plasmid pDR274), (c) hU6T7 hybrid promoter containing both the human U6 and T7 bacteriophage promoters, (d) U6T7G hybrid promoter with the A nucleotide removed from the +3 position (e) U6T7 hybrid promoter with C24 sequence, (f) U6T7G hybrid promoter lacking a G nucleotide at the +2 position with C24 sequence, (g) pX330 based hU6 promoter with C24 sequence and GG at the +1 /+2 position, (h) pX330 based hU6 promoter with C24 sequence, (i) pDR274 T7 promoter with C24 sequence. TATA marks the hU6 TATA box; +1 marks the transcription start site (G); double underlined sequence (a-d) are 4 base pair cloning overhangs generated by restriction endonuclease BsaI in T7-based and U6T7 vectors or restriction endonuclease BbsI in hU6-based expression vectors. Recognition sites for BsaI and BbsI are indicated in bold; single underlined sequence, C24 target template.

The pU6T7G vector was made by removing the BsaI stuffer fragment and inserting the annealed oligonucleotides (Sigma): U6T7GT, 5’-TAGGGAGACCGAGAGAGGGTCTCA-3’ and U6T7GB, 5’-AAACTGAGACCCTCTCTCGGTCTC-3’. This construct retains the BsaI cloning sites but the 5’ cloning site is now ATAG, with the G being the first nucleotide transcribed. Sequences of these vectors are shown in Fig 1. pU6T7 (plasmid #71462) and pU6T7G (plasmid #71463) were deposited with Addgene.

### Cloning gRNA target sequence into expression vectors

The C24 gRNA target sequence, 5’-ACTCCAGTCTTTCTAGAAGA(TGG)-3’ (UCSC Genome Browser GRCm38/mm10 chr6:113076028–113076047 (-)), was cloned into pU6T7 and pU6T7G plasmid vectors. This C24 gRNA sequence lies in intron 1 of the ROSA26 gene locus. The last three nucleotides (TGG) at the 3’- end is the PAM motif. The C24 template was also cloned into pX330-U6-Chimeric_BB-CBh-hSpCas9 vector (plasmid # 42230 from Feng Zhang, obtained from Addgene) with either one G (pX330-C24G) or two Gs (pX330-C24GG) at the hU6 transcriptional start site. The C24 sequence was also cloned into pDR274 (plasmid # 42250 from Keith Joung, obtained from Addgene), which contains only a T7 promoter. For cloning into U6T7, U6T7G and pDR274 vectors, a complementary C24 oligonucleotide was made (Sigma) with BsaI restriction enzyme cohesive ends. To clone the C24 gRNA template into the pX330 vector, a C24 oligonucleotide with cohesive ends compatible with the BbsI cloning site was used [10]. Sequences of these vectors were confirmed by Sanger sequencing prior to experimental use (Fig 1).

### Production and testing of gRNA for site-specific DNA cleavage

Plasmids expressing gRNA together with the hCas9 plasmid (plasmid # 41815 provided by George Church, obtained from Addgene) were transfected into NIH3T3 cells using Lipofectamine2000 (Life Technologies). As a control, the pX330-U6-Chimeric_BB-CBh-hSpCas9 plasmid and the pDR274 plasmid were also transfected into NIH3T3 cells. After three days of culture, cells were harvested and genomic DNA prepared using a Qiamp DNA mini kit (Qiagen). To detect CAS9-induced DNA cleavage, PCR was performed using primers flanking the C24 target locus. Amplified products were denatured and slowly annealed, then treated with T7 Endonuclease I (NEB) and analyzed by gel electrophoresis [11]. A control for non-specific DSB induced by CAS9, the FOS gene locus was also examined. Primers used for PCR amplification of the target region in the Rosa26 locus are: ROSA26G, 5’-AGTGTTGCAATACCTTTCTGGGAG-3’ and ROSA26H, 5’-GGCGGATCACAAGCAATAATAACCTG-3’. Primers used for PCR amplification of the Fos locus are: FOS-F, 5’-GGCTGGCCCTGTATTCCTGAT-3’ and FOS-R, 5’-TCTTCTGACCCTTCCCTACTGAGC-3’

To synthesize gRNA *in vitro*, expression vectors were first linearized by digestion with DraI, treated with proteinase K, deproteinized by extraction with phenol:chloroform and precipitated. The purified vector DNAs were then used as templates for *in vitro* transcription. Using the MEGAshortscript T7 kit (Life Technologies), gRNAs were synthesized *in vitro* and purified with the MEGAclear kit (Life Technologies). pCAG-T3-hCAS-pA vector (plasmid # 48625, provided by Wataru Fujii & Kunihiko Naito, obtained from Addgene) was modified to contain a T7 promoter upstream of Cas9 and is designated pCAG-T3-T7-hCAS-pA (P. Romanienko, unpublished data). The pCAG-T3-T7-hCAS-pA vector was linearized with SphI, purified and CAS9 mRNA was synthesized using the T7 mMessage mMachine Ultra kit (Life Technologies) but omitting the polyadenylation step. The CAS9 mRNA was then purified using the Rneasy Cleanup kit (Qiagen). To test for DNA cleavage activity *in vitro*, Rosa26 target DNA template and Fos control DNA template were incubated with gRNA and Cas9 protein (NEB) following the protocol provided by the vendor (NEB). Template DNA was amplified by PCR and purified using a QIAquick PCR Purification kit (Qiagen). After incubation of the synthetic template with gRNA/CAS9, cleavage at the target locus was analyzed as described above.

### Microinjection of gRNAs into fertilized mouse eggs

To test for gene editing activity in mice, CAS9 mRNA was mixed with gRNA at a final concentration of 25 ng/ul and 50 ng/ul, respectively, and injected into the pronucleus of B6CBAF2/J fertilized eggs. Three week old B6CBAF1/J females were purchased from the Jackson Laboratory, housed in individually ventilated cages, and maintained on a 14 hour light and 10 hour dark cycle with food and water provided *ad libitum*. After 3–5 days of acclimation, female mice were superovulated, mated with stud males, and embryo harvested according to standard procedure [12]. Injected zygotes were cultured for 3 days and single embryos were lysed in 5 ul of 25 mM NaOH in 0.2 mM EDTA, neutralized with 5 ul of 40 mM Tris, pH 5, using the Hotshot method [13]. Genomic DNA was amplified by PCR for 40 cycles using 1 ul of lysate in a 10 ul Advantage 2 polymerase mix (Clontech) and the presence of DSBs at the target locus was analyzed using a T7 endonuclease digestion assay as described. The animal procedures in this study were approved by and done in accordance with Memorial Sloan Kettering Cancer Center Institutional Animal Care and Use Committee (IACUC) and NIH and USDA guidelines.

## Results

Using CRISPR to perform gene editing in mice requires injection of gRNA and the CAS9 mRNA or protein into the cytoplasm or pronucleus of fertilized eggs. Alternatively, one can inject a plasmid vector containing both the gRNA and the CAS9 genes to achieve the same goal: however, injection of plasmid vector into the pronucleus can result in the integration of the plasmid DNA into the mouse genome [6]. An advantage of injecting RNA is that large quantities of nucleic acid can be introduced into the zygote and this is especially beneficial for multiplex targeting. In either case, microinjection is a labor-intensive process as many eggs need to be injected to obtain sufficient founder mice that possess the desired genetic modification. Therefore, prior to injection, gRNAs are generally first tested in mammalian cell lines with CAS9 to determine the efficiency of DNA cleavage at the target locus. Frequently, multiple gRNAs are tested in order to choose the one with optimal activity for mouse production. For activity testing in cells, gRNAs are expressed from a vector containing an hU6 promoter. For the purpose of microinjection into zygotes, gRNAs are synthesized *in vitro* from a different vector using a T7 bacteriophage promoter. To simplify this process, a vector that contains a single promoter for *in vitro* transcription and for expression of CRISPR gRNAs in mammalian cell was created.

### Design of the hybrid U6T7 promoter

To utilize an hU6 and a T7 promoter simultaneously, it is necessary to ensure that the initiation of transcription from each promoter is efficient and that they do not interfere functionally with each other. The hU6 promoter can be altered without loss of transcriptional function provided that the distance between the TATA box and the initiation site is maintained [14]. To construct a hybrid hU6 and T7 promoter, the T7 bacteriophage promoter was placed downstream of the hU6 TATA box such that both promoters utilize the same G for initiation (Fig 1). Since transcription initiation from a T7 promoter is greatly facilitated by having a GG dinucleotide at the start site, vectors were designed to possess either a single G (hU6T7G) or a GG dinucleotide (hU6T7) at the start site of the U6T7 hybrid promoter in order to compare the efficiency of gRNA expression (Fig 1). For both vectors, a gRNA template sequence was placed downstream of the hybrid promoter (hU6T7-C24; hU6T7G-C24, Fig 1).

### Transcriptional activity of the U6T7 hybrid promoter in mouse 3T3 cells

**Fig 2:**
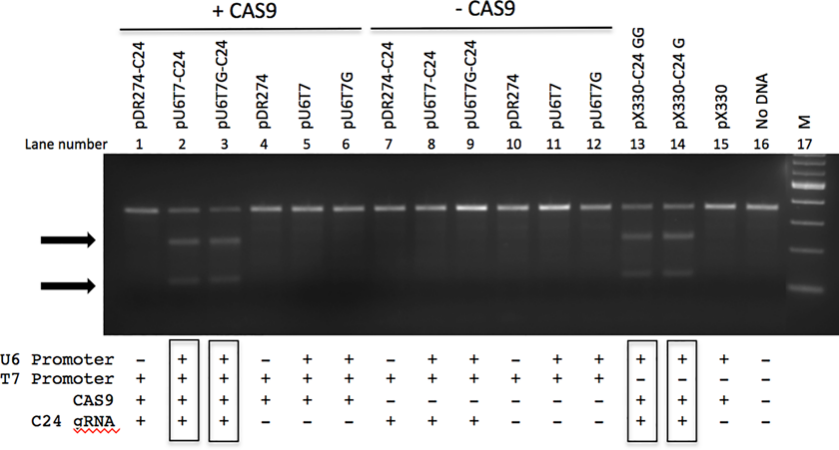
gRNA expressed from the U6T7 hybrid promoter creates INDELs in mouse 3T3 cells. Expression vectors containing the pU6T7 hybrid promoter as well as vectors containing only a T7 promoter (pDR274) or hU6 promoter (pX330) were transfected into NIH-3T3 cells. Vectors containing the ROSA26 target sequence are: pU6T7-C24, pU6T7G-C24, pDR274-C24, pX330-C24 G, pX330-C24 GG. Vectors serving as controls lack the C24 sequence (pU6T7, pU6T7G, pDR274, and pX330). The pU6T7 and pDR274 vectors were cotransfected with a CAS9 plasmid whereas the pX330 vector contains its own CAS9 gene expressed via the CMV promoter. The CAS9 expression vector used for co-transfections is the hCAS9 plasmid. M, 100 bp ladder marker (NEB). + and–indicate the presence and absence of the components in each plasmid vector, respectively,. Boxed indicators represent 3T3 cells with INDELs detected at the ROSA26 locus. Arrows indicate T7 endonuclease cleavage products. Non-transfected cells were also assayed for INDELs (Lane 16).

To verify that the modified hU6 hybrid promoter is functional in mammalian cells, the C24 gRNA sequence was chosen to test the ability of the CRISPR to target the Rosa26 locus in the mouse genome. Short 5’ extensions of gRNA sequences have been shown to be well tolerated, causing no significant change to gRNA specificity or activity [15]. When co-transfected into mouse 3T3 cells with a CAS9 expression vector (hCas9), the U6T7 hybrid promoter is capable of expressing a gRNA that can catalyze a DSB at the ROSA26 locus as indicated by the presence of INDELs at the target site (Lanes 2 and 3, Fig 2). In the absence of CAS9, INDELs were not detected (Lanes 7 and 8, Fig 2). The pDR274-C24 vector (Fig 1), which contains only a T7 promoter and the C24 gRNA template, did not produce detectable INDELs in 3T3 cells, suggesting that gRNA was not produced (Lane 5, Fig 2). Expressing the C24 gRNA and CAS9 mRNA from the pX330 vector produced INDELs whether the gRNA template had one (pX330-C24G, Fig 1) or two G nucleotides (pX330-C24GG, Fig 1) at the initiation site (lanes 13 and 14, Fig 2). As expected, expression of CAS9 alone did not produce DNA cleavage at the target locus (Lane 6, Fig 2).

### Transcriptional activity of the U6T7 hybrid promoter *in vitro*

**Fig 3:**
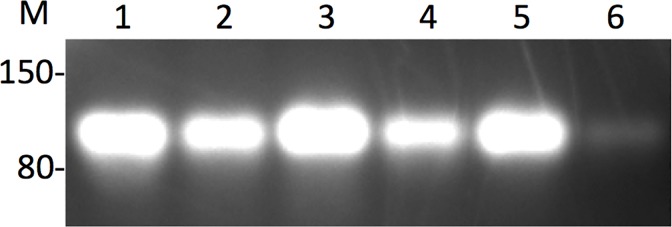
RNAs transcribed from the U6T7/U6T7G hybrid promoter. Lane 1: DR274; Lane 2: DR274-C24; Lane 3: pU6T7, Lane 4: pU6T7-C24; Lane 5: pU6T7G; Lane 6: pU6T7G-C24. M, RNA size marker, 150 nt and 80 nt. Each lane contains 2.5 ul of purified in vitro transcribed RNA (50 ul total elution volume).

Synthesis of gRNA *in vitro* was performed as described in Material and Methods. As shown in Fig 3, most of the expression vectors produced significant amounts of RNA with the exception of the pU6T7G-C24 vector (Fig 3) that has only one G at the transcription start site. All other templates have G at both the +1 and +2 positions. The yield of RNA for each construct is as follows: pDR274, 80 ug; pDR274-C24, 62 ug; pU6T7, 101 ug; pU6T7-C24, 49 ug; pU6T7G, 110 ug; pU6T7G-C24, 13 ug.

**Fig 4:**
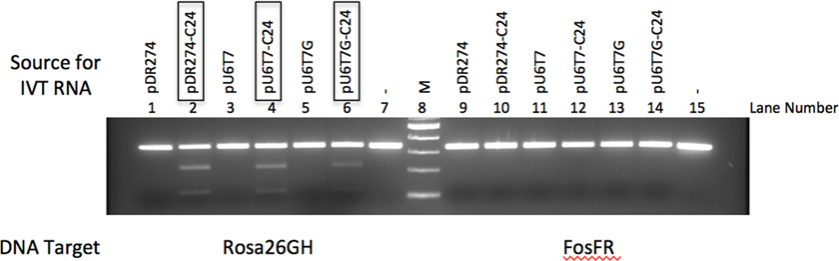
gRNA produced by *in vitro* transcription from the U6T7 hybrid promoter directs cleavage of target DNA. gRNA were produced by *in vitro* transcription from the T7 promoter in the pU6T7 or the pDR274 vectors. The gRNAs, in the presence of CAS9 protein (NEB), cleaves the ROSA26 target site *in vitro*. The DNA template provided to test for CRISPR activity is a PCR product obtained from amplification of the ROSA26 gene locus. As a control, the mouse Fos gene amplicon was used as a template. Expression vectors with gRNAs containing the ROSA26 target sequence are: pU6T7-C24, pU6T7G-C24, pDR274-C24. Expression vectors lacking the ROSA26 template sequence are: pU6T7, pU6T7G, pDR274. Lanes 7 and 15 are the PCR amplicons containing ROSA26 and Fos gene target sequences, respectively. M, DNA size marker (NEB). Boxed vectors identify the transcribed gRNAs that produced INDELs at the target site after incubation with CAS9 protein. Arrows indicate the T7 endonuclease assay products.

All three *in vitro* transcribed C24 gRNAs cleaved the ROSA26 PCR amplicon when CAS9 protein is provided in *trans* (Fig 4, lanes 2, 4, 6). The RNAs obtained from *in vitro* transcription of empty vectors had no effect on the same ROSA26 target (Fig 4, lanes 1, 3, 5). When the same gRNAs were tested on a PCR amplicon derived from the mouse fos gene, no DSB was observed, thus providing evidence of specificity of gRNA-directed cleavage (Fig 4, lanes 9–14).

### Functional activity of gRNAs expressed from pU6T7 vectors in embryos and mice

The gRNAs obtained from *in vitro* transcription of hybrid promoters were coinjected with CAS9 mRNA into the pronuclei of fertilized mouse eggs. One round of injection was performed for each gRNA shown in Table 1. The injected zygotes were cultured for 3 days and then analyzed for the presence of INDELs at the ROSA26 locus using the T7 endonuclease digestion assay (S1 File, S1 Fig, S2 Fig, S3 Fig, S4 Fig, S5 Fig). For each gRNA containing the C24 recognition sequence, INDELs were seen at similar frequencies indicating that both the pU6T7 and the pU6T7G vectors produced fully functional, target-specific gRNAs. Specific cleavage at the ROSA26 locus was not detected when RNAs made from vectors lacking the C24 sequence (U6T7, U6T7G) were used.

**Table 1:** Frequency of INDEL formation in mice using gRNA synthesized from pU6T7 expression vectors.

RNA Injected	# zygotes injected	# embryos screened*	# embryos* with indels	INDEL frequency
**DR274-C24**	32	30	23	77%
**U6T7**	34	32	0	0%
**U6T7-C24**	30	21	17	80%
**U6T7G**	42	32	0	0%
**U6T7G-C24**	40	39	29	74%

gRNAs were synthesized from either the U6T7/U6T7G hybrid promoters or from the T7 promoter. CAS9 mRNA were co-injected with the gRNAs into fertilized mouse eggs.

* Not all injected zygotes developed to blastocysts after 3 days of culture and results are compiled only from blastocysts and morulae containing 8 or more blastomeres. In some cases, the PCR failed to yield any products and these were also excluded from our analysis.

In 2015, our Core used the pU6T7 vector to generate more than 60 different gRNAs for testing in tissue culture cells and most, if not all, produced INDELs in the target loci (data not shown). In addition, gRNAs transcribed *in vitro* from the pU6T7 vector were used to perform twelve gene editing projects in mice and these generated successfully INDELs, deletions and other gene modifications at the endogenous target loci desired (data not shown).

## Discussion

The testing of gRNAs in tissue culture cells prior to zygote injection to generate genetically modified animals is a prudent step for any laboratory to undertake especially for a Core facility where resources are limited. Those gRNAs that show weak or no cleavage activity in cells can be excluded from microinjection thereby increasing the frequency of obtaining founder animals with the appropriate genetic alteration. Since cell transfection assays require gRNAs expressed from an hU6 promoter while gRNAs for microinjection are transcribed from a T7 promoter *in vitro*, two expression plasmids are needed to achieve these goals. To simplify the procedure in obtaining gRNAs for both cell transfection assays and zygote injections, we designed and constructed a vector that contains an hU6 and T7 hybrid promoter that is capable of expressing gRNA in cells as well as transcribing gRNA *in vitro*.

The hU6 based vectors require a single G to initiate transcription, while RNAs produced from the T7 bacteriophage promoter usually have 2 G nucleotides or a G at +1 followed by a purine at its 3’-end for optimal expression. That the single G in the U6T7G-C24 vector supports transcription by T7 polymerase but produced a lower absolute amount of RNA tends to support this notion (lane 4, Fig 3 and Table 1). Extensions of the 5’ end of gRNAs tend not to affect their function, therefore, gene editing ability should remain the same. A direct comparison between RNA produced from hU6 and U6T7 hybrid promoters showed little difference as monitored by DNA cleavage at the ROSA26 locus in mouse 3T3 cells (Table 1). Although the amount of RNA obtained from *in vitro* transcription can vary by 3–5 fold, sufficient RNA is produced for injection into mouse zygotes. While other U6 and T7 promoter fusion configurations are possible, the present U6T7 hybrid promoter maintains the G at the cognate position for optimal transcription from the hU6 promoter (Fig 1). The advantage of using an expression vector with a bifunctional promoter is evident where genome manipulation of animals using RNA is preferred [16]. Since only one vector is required for expressing gRNAs in cells to monitor for activity and to produce gRNA for injection into zygotes, this simplifies the process of CRISPR/CAS mediated genome editing in animals. Furthermore, the time and financial resources saved by using a single expression vector when large numbers of gRNAs are made can be substantial. For example, in 2015, our Core tested at least sixty gRNAs in cells prior to initiating zygote injections to make the gene edited mice requested by investigators. Therefore, most Core facilities will find this U6T7 expression vector to be of great benefit.

## Supporting Information

S1 Fig Blast-Gel2. (TIF)

S2 Fig Blast-Gel3. (TIF)

S3 Fig Rosa-BlastT7A. (TIF)

S4 Fig Rosa-BlastT7B. (TIF)

S5 Fig Rosa-BlastT7C. (TIF)

S1 File Rosa26 (C24) T7EI assay results gel summary. (XLSX)
