# Supplementary figures and images for: A Vector with a Single Promoter for In Vitro Transcription and Mammalian Cell Expression of CRISPR gRNAs
Source: PLoS One. 2016 Feb 5;11(2):e0148362. doi: 10.1371/journal.pone.0148362 (PMC4744079; doi:10.1371/journal.pone.0148362)

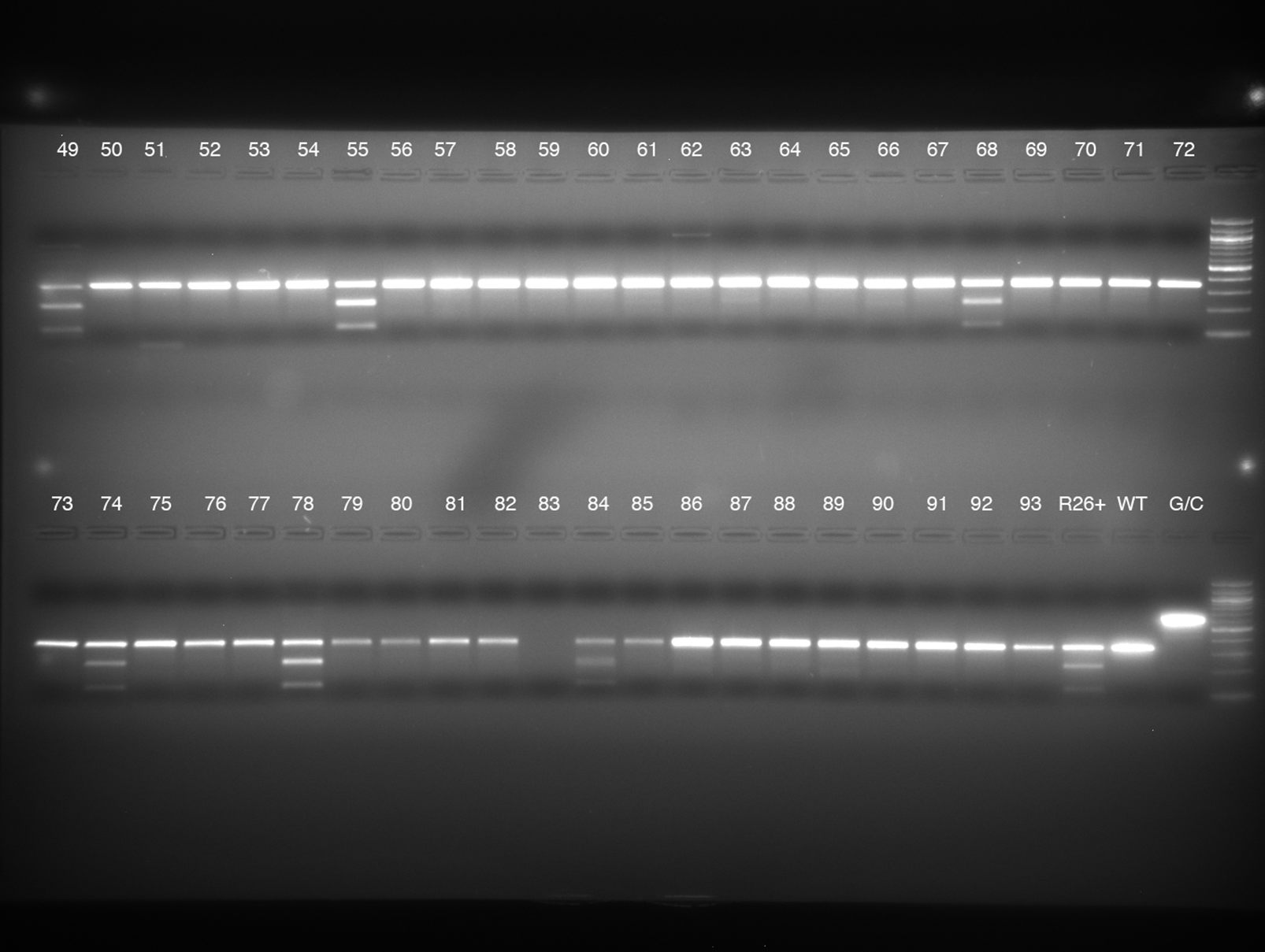

Supplement: S1 Fig — (TIF) [file pone.0148362.s001.tif]

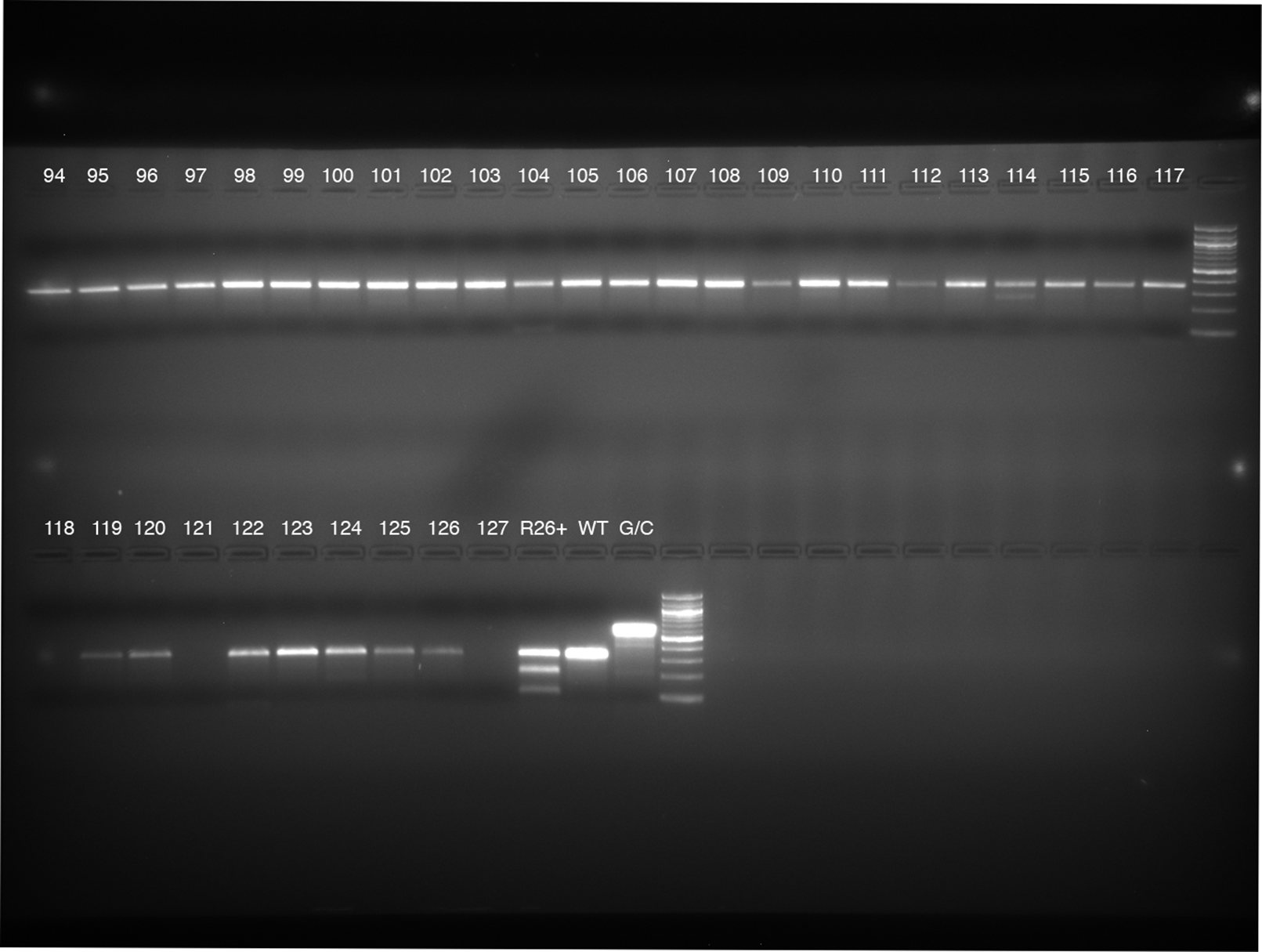

Supplement: S2 Fig — (TIF) [file pone.0148362.s002.tif]

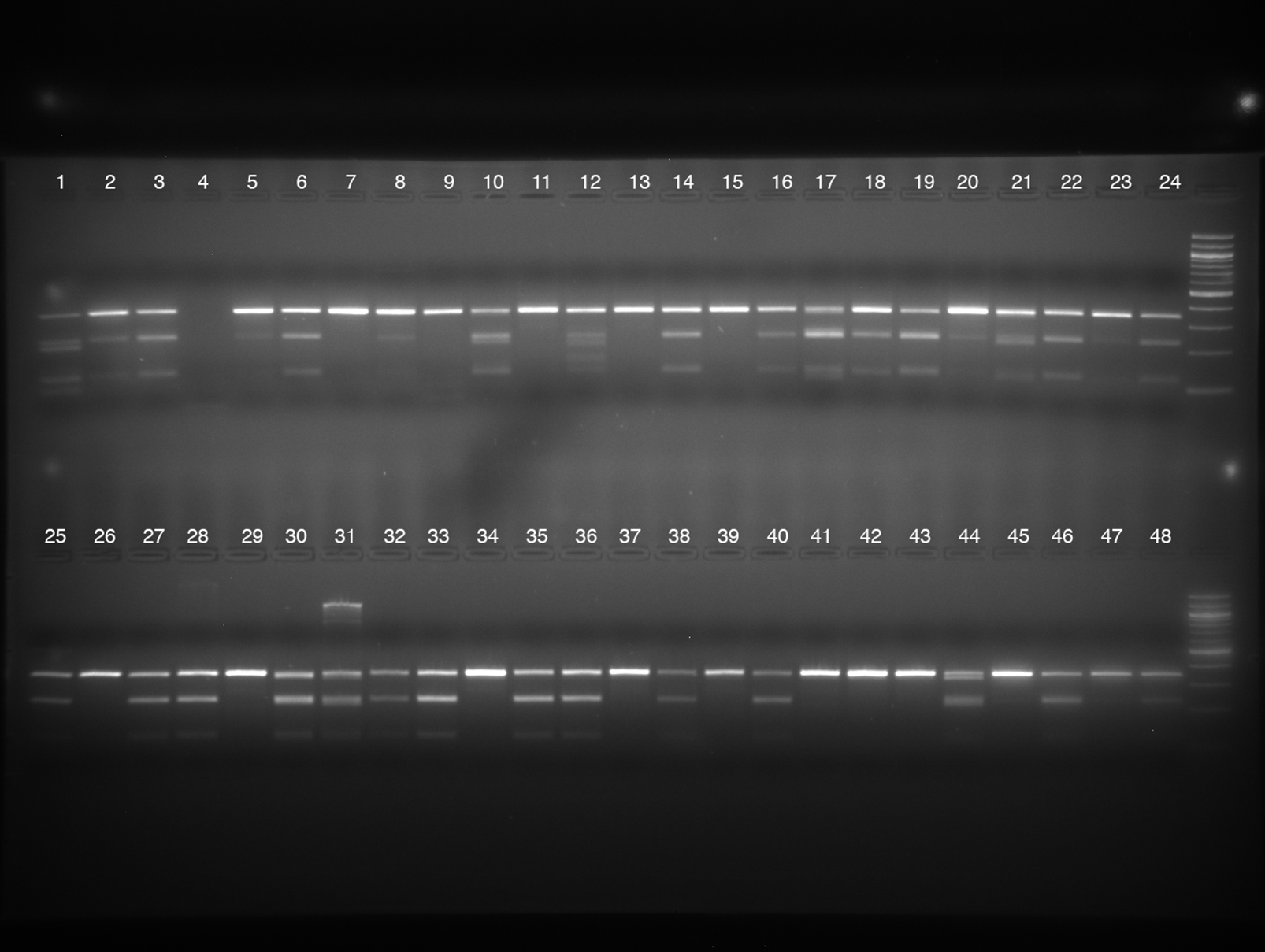

Supplement: S3 Fig — (TIF) [file pone.0148362.s003.tif]

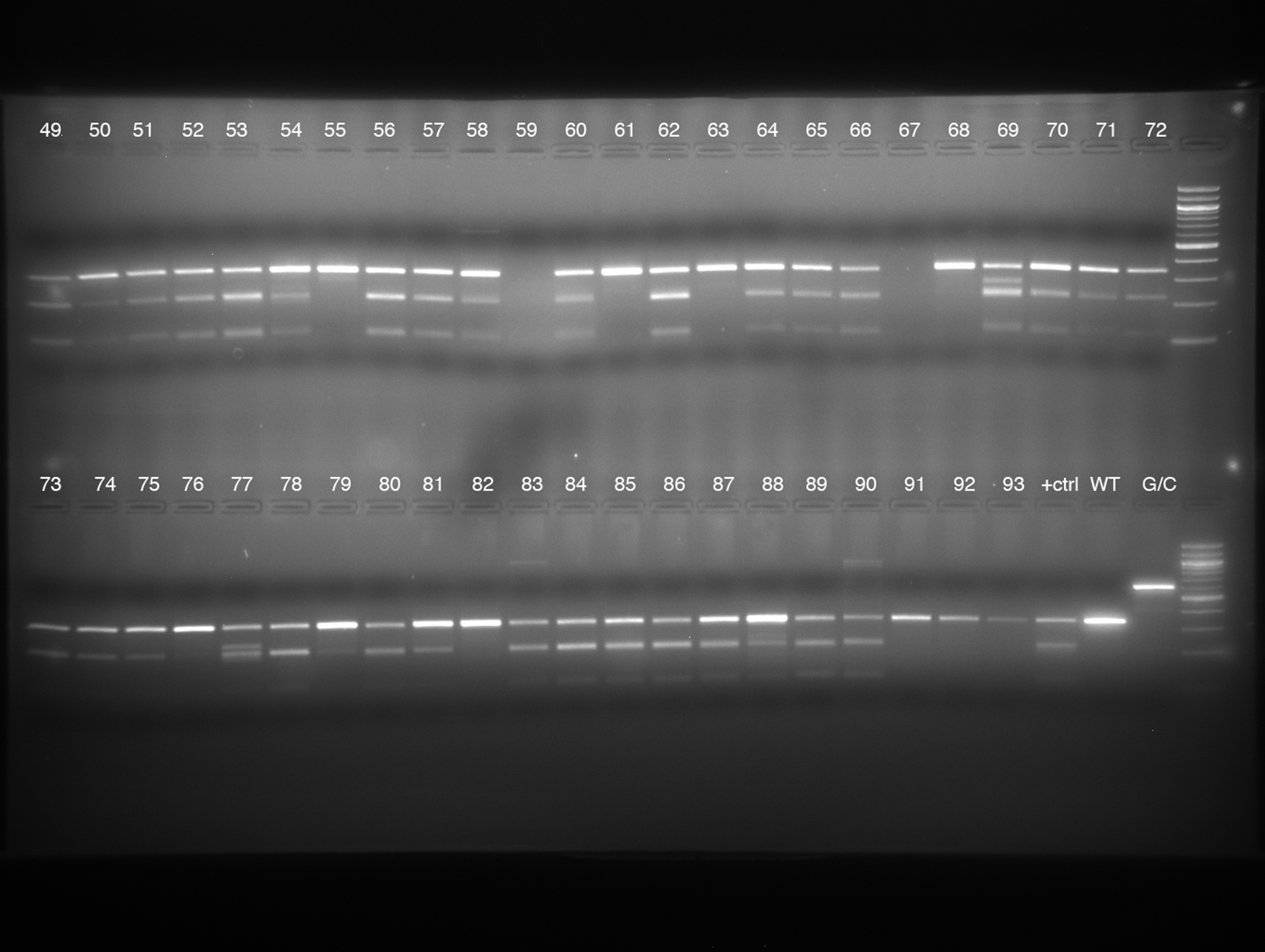

Supplement: S4 Fig — (TIF) [file pone.0148362.s004.tif]

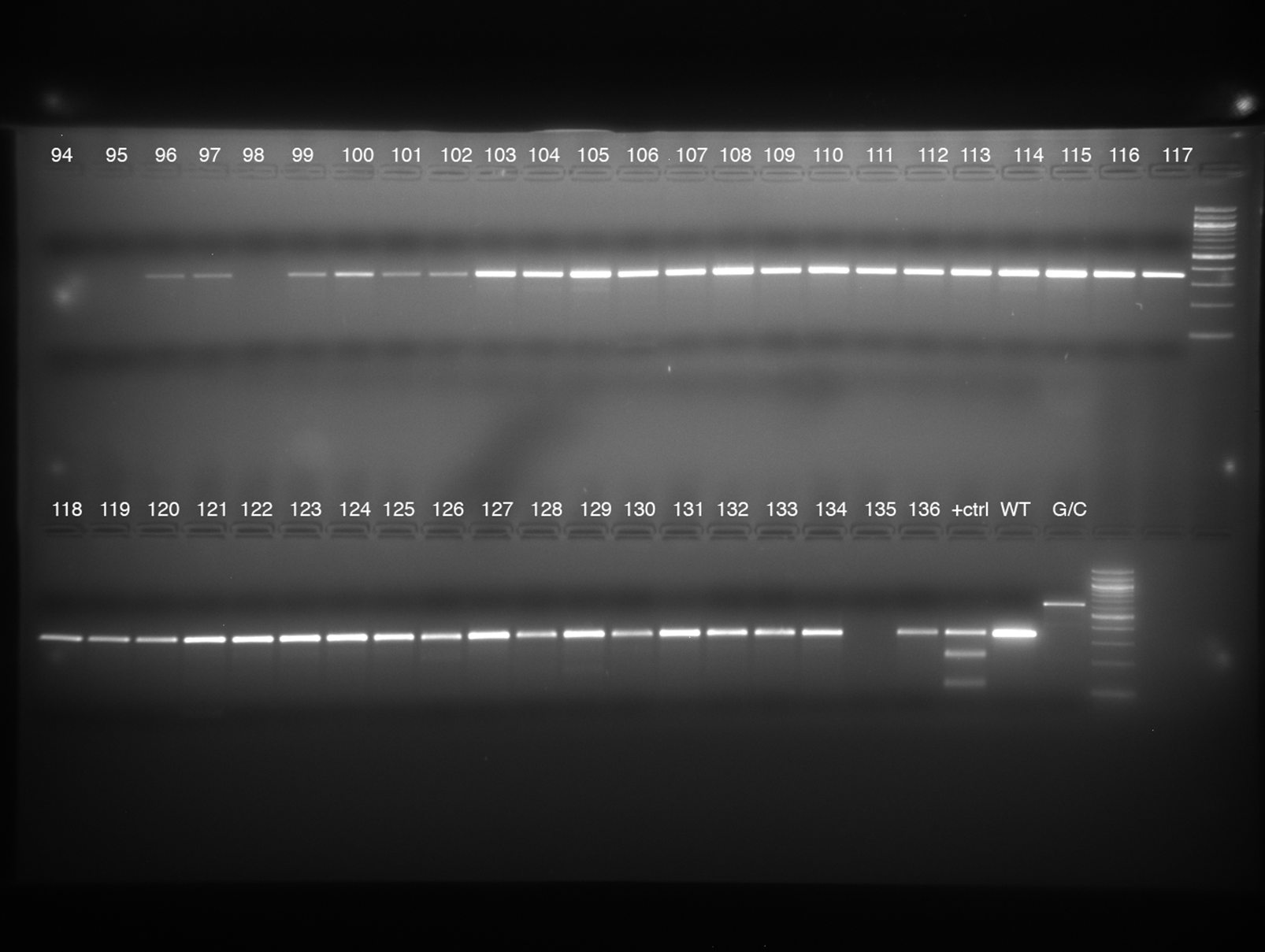

Supplement: S5 Fig — (TIF) [file pone.0148362.s005.tif]
